# Personalized Medicine Approaches in Prostate Cancer Employing Patient Derived 3D Organoids and Humanized Mice

**DOI:** 10.3389/fcell.2016.00064

**Published:** 2016-06-23

**Authors:** Monica Bartucci, Anna C. Ferrari, Isaac Yi Kim, Alexander Ploss, Martin Yarmush, Hatem E. Sabaawy

**Affiliations:** ^1^Rutgers Cancer Institute of New Jersey, Rutgers UniversityNew Brunswick, NJ, USA; ^2^Department of Molecular Biology, Princeton UniversityPrinceton, NJ, USA; ^3^Center for Engineering in Medicine, Shriners Hospitals for Children and Department of Surgery, Massachusetts General Hospital, Harvard Medical SchoolBoston, MA, USA; ^4^Department of Biomedical Engineering, Rutgers UniversityNew Brunswick, NJ, USA; ^5^Department of Medicine, Rutgers Biomedical and Health Sciences (RBHS)-Robert Wood Johnson Medical School, Rutgers UniversityNew Brunswick, NJ, USA

**Keywords:** organoids, prostate cancer, precision medicine, precision therapeutics

## Abstract

Prostate cancer (PCa) is the most common malignancy and the second most common cause of cancer death in Western men. Despite its prevalence, PCa has proven very difficult to propagate *in vitro*. PCa represents a complex organ-like multicellular structure maintained by the dynamic interaction of tumoral cells with parenchymal stroma, endothelial and immune cells, and components of the extracellular matrix (ECM). The lack of PCa models that recapitulate this intricate system has hampered progress toward understanding disease progression and lackluster therapeutic responses. Tissue slices, monolayer cultures and genetically engineered mouse models (GEMM) fail to mimic the complexities of the PCa microenvironment or reproduce the diverse mechanisms of therapy resistance. Moreover, patient derived xenografts (PDXs) are expensive, time consuming, difficult to establish for prostate cancer, lack immune cell-tumor regulation, and often tumors undergo selective engraftments. Here, we describe an interdisciplinary approach using primary PCa and tumor initiating cells (TICs), three-dimensional (3D) tissue engineering, genetic and morphometric profiling, and humanized mice to generate patient-derived organoids for examining personalized therapeutic responses *in vitro* and in mice co-engrafted with a human immune system (HIS), employing adaptive T-cell- and chimeric antigen receptor- (CAR) immunotherapy. The development of patient specific therapies targeting the vulnerabilities of cancer, when combined with antiproliferative and immunotherapy approaches could help to achieve the full transformative power of cancer precision medicine.

Although adult organs are three dimensional (3D), our ability to understand tissue development, function, and cellular patho-physiology has mainly relied on knowledge from flat two-dimensional (2D) cell culture studies. With more knowledge gained, new approaches have been developed, for growing both normal and diseased tissues, allowing the creation in a dish of organ-specific mini 3D structures termed “organoids” (Lancaster and Knoblich, 2014). Organoids have been generated from both pluripotent stem cells (PSCs) and adult stem cells (SCs) by mimicking the cellular and tissue developmental processes (Yin et al., 2016). These organoids are believed to stem from single multipotent SCs or organ progenitors capable of differentiation and self-organization to form structures morphologically and functionally resembling the corresponding *in vivo* organ (Cukierman et al., 2002; Birgersdotter et al., 2005; Griffith and Swartz, 2006; Nelson and Bissell, 2006). Thus, the 3D organoid model offers a vast range of attractive employments into the biomedical field.

The knowledge around the capacity of an individual cell to form a whole organism dates back to the early 1900, with the discovery of self-organized dissociated sponge cells that are able to rear an entire multi-cellular animal (Wilson, 1907). Since then, organoid culture systems have been established with the aim of supporting SC engineering applications and, lately, for the utilization in examining personalized therapeutic approaches and validating targets for cancer therapy (Bissell and Labarge, 2005; Postovit et al., 2007; Hynds and Giangreco, 2013). For the latter, there is currently a strong need to explore and validate the utilization of patient derived organoids for the developing science of precision medicine. To achieve this goal, it is essential to point out that when it comes to applying precision medicine principles to the oncology practice, there is a widespread tendency to underestimate the complexities of cancer as a genetic disease, for instance assuming that targeting specific genomic vulnerabilities will correspond with therapy effectiveness. Nevertheless, if genomic expression profiling helps to narrow down and uncover a certain number of deregulated gene patterns, whose expression varies between different cell/patient populations, and drive aberrant cancer pathways, transcriptional profiling might not fully correlate with the functional state of the encoded cellular proteins. Moreover, profiling does not provide information on protein-protein interactions (Hunter, 2000; Celis and Gromov, 2003), and most critically, nor does it fully account for the extreme clonal heterogeneity recently revealed (Barbieri et al., 2013). In this regard, the possibility to functionally test treatment protocols directly on patient tumor cells, before therapy implementation, in an “*in vivo* mimicking” environment, and preferably by utilizing single cell assays (Ryan et al., 2011), or at a minimum by performing studies at the clonal level (Sabaawy, 2014), would definitely address these limitations. Here, we present the key strengths, weaknesses, and possible applications of the 3D organoid models into the biomedical field, with a particular emphasis on the prostate cancer (PCa) therapeutic arena.

## Organoids in prostate cancer

Advanced and metastatic PCas are common causes of cancer related death in Western men (Siegel et al., 2015). They are characterized by a remarkable genomic complexity mirrored in the clinically variable behavior of the disease (Berger et al., 2011). In the human body, PCa is sustained within a complex organ-like structure whose survival and function relies upon the dynamic interactions of tumor cells with stromal, endothelial, immune cells and the surrounding extracellular matrix (ECM; Moore and Lemischka, 2006). Despite its incidence, PCa has proven very difficult to propagate *in vitro* and the lack of preclinical models that reproduce the cellular diversity of the tumor niche and the interactions with the ECM has hampered advancements toward interrogating disease progression and therapeutic responses (Izumchenko et al., 2016). For instance, cell lines solely grown in monolayers lack patients' PCa heterogeneity (Wilding and Bodmer, 2014). Moreover, although multi-allelic genetically engineered mouse models (GEMM) contribute to the understanding of different phases of tumorigenesis, they fail to mimic PCa progression or to reproduce the diverse mechanisms of therapy resistance (Meads et al., 2009). On the other hand, patient derived xenografts (PDXs) while more representative of PCa are difficult to establish, expensive, time consuming, tumor clones undergo selective engraftments, and lack immune regulation (Garrido-Laguna et al., 2012; Julien et al., 2012; Cassidy et al., 2015). The human prostate is characterized by glandular epithelial/diverse cell organization. When prostatic tissues are maintained in culture and embedded in a complex matrix environment, this 3D culture system would more closely reflect the natural *in vivo* behavior of PCa tissue, rendering its *in vitro* propagation easier and its utilization for prediction of therapeutic response much more reliable.

## Human prostate 3D culture

*Ex vivo* culture of the human prostate tissues were originated in the 1970s following the culture of mouse prostate tissues (Pretlow et al., 1995). A variety of culture methods have since been developed involving the use of tissue slices and culture over various scaffolds. These different culture methods have been shown to allow benign prostatic tissue viability and maintenance for about a week, although the culture of human PCa tissues is generally not successful using these approaches (Centenera et al., 2013). To date, human PCa organoids have only been generated from patients with advanced disease (Gao et al., 2014). However, molecular studies have clearly established that the primary malignancy is the reservoir of resistant clones causing waves of metastatic seeding to distant sites (Haffner et al., 2013; Gundem et al., 2015). Indeed, while primary PCa is multifocal with extensive clonal heterogeneity (Berger et al., 2011; Fontugne et al., 2016), lethal metastatic disease is mono- (or oligo-) clonal only acquiring subclonal heterogeneity from adaptations to the microenvironment and/or from the selective pressure of therapy (Barbieri et al., 2013), which might mask fundamental elements of disease progression. Although a 3D organoid system derived from the later stages of the disease may help progress toward developing drugs to control secondary lesion growth and improve the quality of life of patients, identifying the subset of localized PCa foci that are responsible for tumor progression is the key to a more widely successful treatments. Thus, envisioning an efficient culture system to generate single cell-derived PCa organoids from the localized disease, that mirror patients' genomic alterations, would allow the production of personalized biological systems amenable not only to measure treatment agents activity in real time, but also, more importantly, to identify those with highest potential for efficacy in the clinic. This suggests that the opportunity exists to make dramatic improvements in life-extending, possibly curative, therapies by focusing on the assessment of the vulnerabilities of the heterogeneous primary tumor sites.

## Generating organoids from patient derived cells

**Figure 1:**
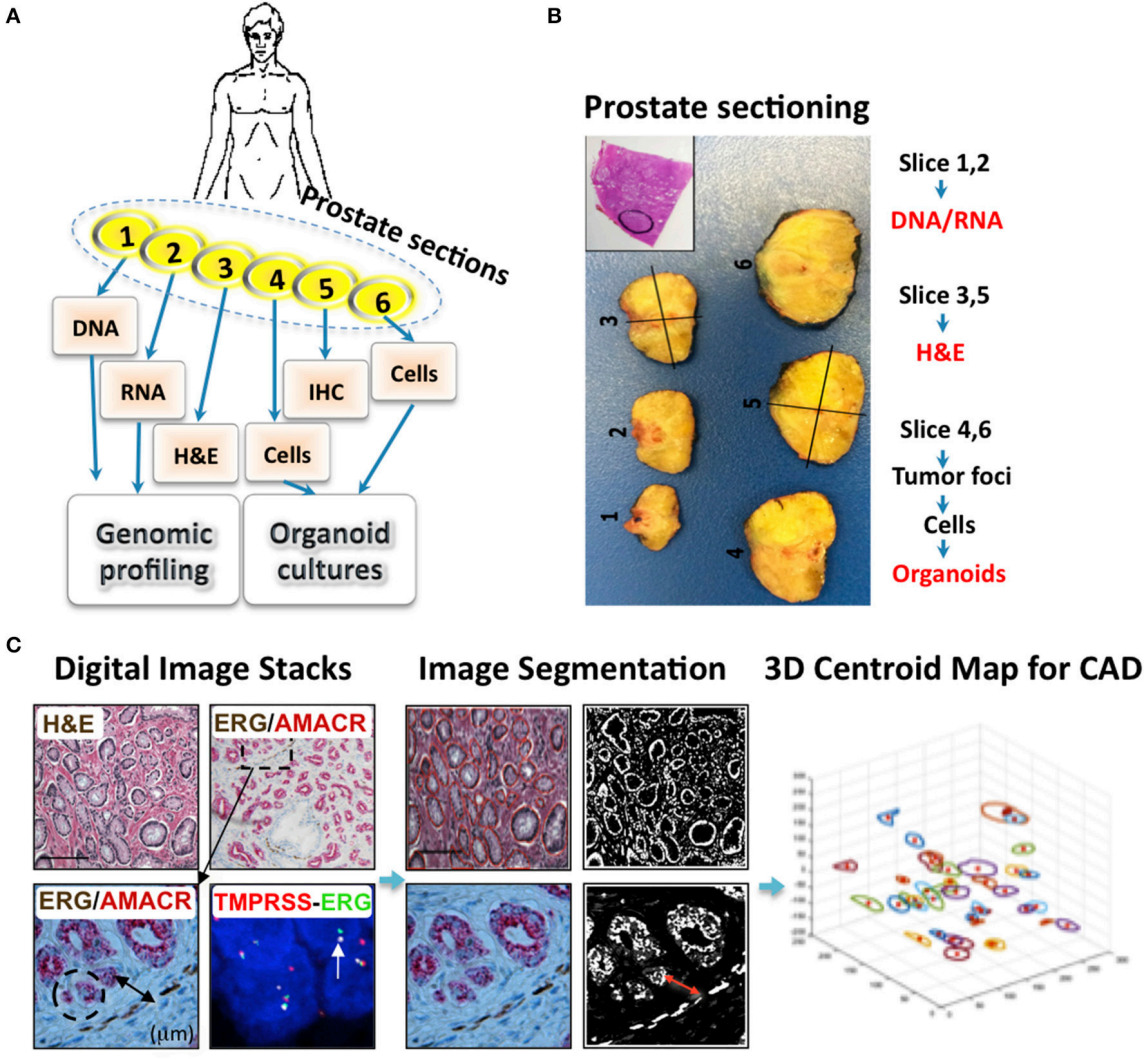
**An interdisciplinary approach for utilizing patient derived organoids for prostate cancer precision therapy. (A)** Patients with localized and metastatic PCa were enrolled after an informed consent. Patients underwent first line treatment, and then had radical prostatectomy (RP) and biopsy of metastatic (mets) lesions. Next, Patients' tissues from RP and mets were used for generating personalized organoids. The prostate gland was examined by an experienced Pathologist to identify PCa foci, and under aseptic conditions divided into six sections. Sections 1 and 2 are used for DNA and RNA for sequencing to define PCa subgroups. Sections 3 and 5 are used for PCa foci mapping based on Hematoxylin and eosin (H&E) and IHC staining, and Fluorescent *in situ* hybridization (FISH) for the TMPRSS2-ERG fusion. Slides are scanned into digital images, while mirror sections 4 and 6 are isolated under aseptic conditions and cells are separated for organoid culture. **(B)** Demonstration of prostate gland sectioning. Inset indicates H&E staining with the tumor area mapped. **(C)** H&E, IHC, and FISH representative images from a case used for prostate cancer tissue mapping. The tumor areas were identified based on histological and IHC staining for ERG and AMACR and positivity for the Ets fusion (TMPRSS2-ERG) by FISH. Images were digitally scanned, stacked, and image segmentation was used for PCa mapping. Organoids from normal adjacent tissue (NAT), PCa primary foci, and node or bone metastasis were generated for each patient, based on the collective database information, including tumor foci, vascular density and tumor infiltrating lymphocytes, with computer assisted design (CAD) programs. Genomic profiling of primary, mets and their organoids is done after exome, whole genome sequencing (WGS), and RNA sequencing. PCa organoid response to therapy when examined in patient derived organoids allowed the correlation of organoid response to ongoing first line therapy and selecting for second line, more biologically effective therapy, in order to prevent disease progression and development of therapeutic resistance.

*In vitro* 3D epithelial cell cultures have been used to support growth of mammary epithelium (Stingl et al., 2001), hepatic (Mitaka et al., 1999), and intestinal villi (Sato et al., 2009). By utilizing epithelial and mesenchymal developmental signals in 3D organotypic culture containing ECM, we regenerated single stem cell-derived benign prostatic hyperplasia (BPH) and naïve PCa into 3D organoids comprised of both epithelial and mesenchymal tissues. We first established methods for the processing of PCa tissues to generate organoids for examining the genomic vulnerabilities of each patient's cancer tissues with targeted therapy under IRB-approved protocols. Briefly, immediately after the surgical removal of the prostate, the specimen is retrieved from the operating room, registered and serially sectioned from apex (slice 1) to base (slice 6; Figures 1A–C). Tissues obtained from slices 1 and 2 are utilized for DNA and RNA extraction, respectively, and frozen for subsequent sequencing, genomic profiling and molecular studies. H&E and immunohistochemistry (IHC) sections, from slices 3 and 5, respectively, are examined to identify and select a number of tumor foci based on the expression of PCa-specific biomarkers (Bansal et al., 2014). H&E slides are digitally scanned and images are subjected to foci mapping. Fresh tissues are obtained from the foci in slices 4 and 6 that correspond to the mapped tumor areas; cells are isolated and subjected to organoid formation (Bansal et al., 2014). Prostate organoids are coded, retrospectively matched with database record of mapped tumor foci and genomic profiles then examined for therapeutic responses (Sabaawy et al., 2015).

**Figure 2:**
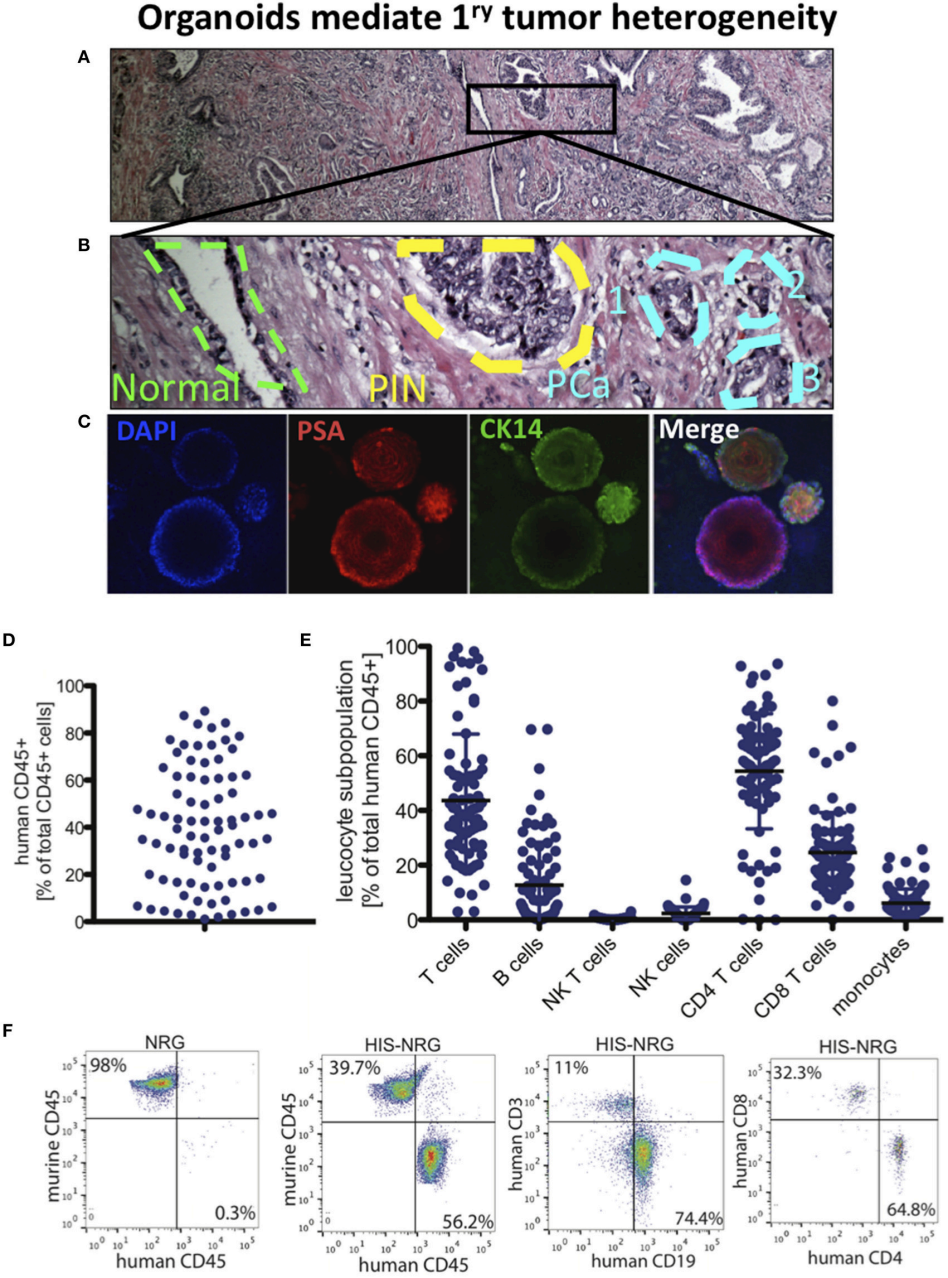
**PCa organoids to model tumor heterogeneity and develop immunotherapy in humanized mice. (A)** H&E of RP section from a PCa patient shown in 4x. **(B)** The outlined area in **(A)** is displayed in 200x, showing the outline of benign prostate gland (green), prostatic intraepithelial neoplasia (PIN) region (yellow), and a three foci region of PCa (Blue). **(C)** Single-cell organoids reflect the heterogeneity in primary PCa. Immunofluorescence (IF) images show DAPI as nuclear staining, PSA (center region), CK14 (in cells lacking PSA staining, i.e., transit amplifying cells). Multiple organoids derived from the same patient's PCa expressing PSA and CK14 (right), low and localized (top) and low/negative (bottom). **(D)** Human immune system (HIS) reconstitution in NRG HIS. Fraction of human CD45+ cells of total CD45+ cells detected in the HSC transplanted NRG mice. **(E)** Indicated leukocyte subpopulations were determined by FACS analysis of PBMC in NRG mice. **(F)** Indicated immune cell subpopulations in NRG and HIS-NRG mice are shown as dot plots.

In proof-of-concept studies, each patient-derived prostate organoids recreated the cellular phenotypic profile of the parental cell of origin in the primary PCa tissue. We matched the normal, prostatic intraepithelial neoplasia (PIN) and PCa foci with regions of normal glands, PIN and PCa in the patient's H&E stained and mapped section (Figures 2A,B) along with marked organoids for cytokeratins (CKs) and prostatic specific antigen (PSA). The prostate epithelium is composed of basal cells, including SCs and transient amplifying (TA) cells, terminally differentiated luminal cells and rare neuroendocrine cells. These cells can be distinguished based on the expression of a variety of markers (Collins and Maitland, 2006). Most basal cells express CK5/14, p63, CD44, CD49, and do not express CK8/18 or PSA. Luminal cells express relatively high levels of PSA and CK8/18, while TA (intermediate) cells co-express basal and luminal markers (Collins and Maitland, 2006). In contrast, PCa diagnosis typically relies on lack of expression of basal cell markers (Prins and Putz, 2008; Bansal et al., 2014). Organoids derived from cells of mirrorimage tissue showed the expected CK/PSA staining that distinguishes normal, PIN and PCa (Collins and Maitland, 2006; Prins and Putz, 2008; Bansal et al., 2014). For instance, PSA^−∕low^ staining in cells that are CK14^+^ represents organoids from TA cells. Smaller organoids with a similar number of PSA^+^ and CK14^+^ cells were derived from a normal gland. Organoids with higher PSA than CK14 represent basal cell derived PIN, while larger organoids with PSA^hi^/CK14^−∕low^ cells were PCa organoids (Figure 2C).

In these studies, we first derived organoids from the bulk of tumor cells and found that only a fraction of these cells (~1%) have organoid forming potential. Using 3D chambers of Matrigel, we have generated prostate organoids that have survived for 3–6 months and could be re-derived to generate serial cultures (Bartucci et al., 2015). In one study, we utilized hypoxic conditions to obtain organoids from 21 out of 24 attempted PCa samples with an efficiency of ~87% (Bartucci et al., 2015). Organoids could also be derived from adult tissue SCs (Shamir and Ewald, 2014) or their tumorigenic counterparts (Sato et al., 2011) and we documented that the ability to form clonal single cell-derived prostate organoids was increased in the CD49b^hi^CD29^hi^CD44^hi^ cell subpopulation (TICs) that has stem-like features and *in vivo* tumorigenic potentials (Bansal et al., 2014).

Because organoids can be maintained in culture over time, they offer a unique opportunity to test multiple agents and their associated toxicity and selective pressure on tumor cells, thus avoiding those agents that are ineffective. That said, although undeniably valuable, there are however still important limitations alongside studies and applications of *in vitro* 3D models (Yamada and Cukierman, 2007). For instance, the absence of a vascular environment in a petri dish makes it difficult for organoids to fully grow and mature in a functional tissue/organ/tumor. Furthermore, in the case of cancer organoids, the lack of diverse cell-cell interactions such as epithelial-stromal or epithelial-endothelial or, even more importantly, of an active host immune response, can still influence the experimental outcomes when patient's responses to therapy are considered.

It has been proven that under the appropriate conditions, endothelial, stromal, and cancer cells can collectively be grown yielding an organotypic tumoral niche (Chong et al., 2014), however, this will not resolve the absence of an efficient immune system. These challenges could be overcome with innovative approaches where primary PCa cells, co-cultured three-dimensionally with stromal and endothelial cells, will subsequently be injected into humanized mice engrafted with the same patient's immune cells. As a consequence, personalized and immune precision therapy approaches can then be examined to identify the optimum treatment sequences and monitor therapy-induced clonal selection processes that are frequent causes of relapse (Baca et al., 2013).

## Developing mice with prostate organoids and a human immune system

While immunotherapy has been successfully integrated into the treatment strategies for melanomas and blood cancers, the results for PCa treatment have been disappointing. To date, the only FDA-approved anticancer vaccine is Sipuleucel-T. A Phase III IMPACT (Immunotherapy Prostate AdenoCarcinoma Treatment) trial that included 512 metastatic castration resistant PCa patients showed that Sipuleucel-T improved the overall survival (OS) by ~4 month and reduced the risk to die by only 22% (Kantoff et al., 2010). Alternative immune approaches include utilizing tumor-associated antigens (TAAs) that stimulate cellular or humoral responses and can efficiently eliminate tumor cells in some cases (Mittal et al., 2014), since peptides derived from TAAs can be presented with MHC class I/II by CD8^+^/CD4^+^ T cells, respectively. Adaptive T-cell- and chimeric antigen receptor- (CAR)-mediated immunotherapy approaches have shown recent successes, but there are no platforms to examine these immunotherapy strategies with standard therapy (Schreiber et al., 2011), or in the context of precision medicine. Primary PCa cells are difficult to engraft in conventional immunocompromised mice. We need to utilize highly immunodeficient mice that will not reject PCa xenografts, will allow high engraftment rates, and will support the differentiation and growth of human cells and tissues. This could be achieved with engineered “Humanized” mice (Drake et al., 2012). Therefore, the goal of our ongoing studies is to generate immune humanized mice with implanted prostate organoids to study PCa tumor-immune cell interactions and quantify the effects of PCa therapy on immune responses to tumors as a stepping-stone toward developing patient specific immunotherapy.

## Reconstitution of mice with a humanized immune system

Humanized mice are immunodeficient animals engrafted with human hematopoietic stem cells (HSCs) that give rise to various lineages of human blood cells throughout the life of the mouse (Drake et al., 2012). By simultaneously humanizing the immune system of recipient animals and challenging them with implanted human tumor cells in prostate organoids, the interactions between human immune cells and tumor cells can be interrogated (Chen and Mellman, 2013). Mice engrafted with components of a human immune system (HIS) are routinely generated by engrafting human HSCs isolated from human fetal liver (HFL), BM, or cord blood into highly immunodeficient mouse strains, such as NOD Rag1^−∕−^ IL2Rα^null^ (NRG) mice, NOD SCID IL2Rα^null^ (NSG) or Balb/C Rag2^−∕−^ IL2Rα^null^ (BRG), that support better human hematopoietic cell engraftment (Figures 2D–F). We have pursued improved strategies to enhance the human immune cell reconstitution and function in humanized mice (Billerbeck et al., 2014). A variety of other strategies are also being pursued, including, but are not limited to: the expression of human orthologs of non-redundant cytokines with limited biological cross-reactivity to foster the development of human immune cell lineages which currently do not develop efficiently in conventional humanized mice; expression of human MHC in the absence of mouse MHC to ensure faithful presentation of self- and virally-derived peptides to human T-cells and to reduce graft-versus-host-disease; co-transplantation of HSC donor-matched human thymic cortical epithelium to facilitate proper T-cell selection; the improvement of lymphoid architectural organization, especially in the spleen and lymph-nodes, to allow for adequate T- and B-cell priming; genetic replacement of non-compatible immune cell receptors and chemokines expressed on non-hematopoietically derived cells to improve immune functions such as immune cell trafficking; and the introduction of a human microbiome to account for the effects of species-specific commensals on the immune system (reviewed in Shultz et al., 2012). We are now poised to reconstitute mice with a HIS from PCa patients along with their prostate organoids to examine the personalized immune responses against PCa cells in the presence or absence of PCa therapy to overcome resistance and provide new approaches for cancer therapy.

With the exciting potential that these models offer, it is worth remembering that HIS mice still have remaining challenges. For example, humanized mice are limited by the availability of human tissue donors and the species incompatibility of ligands. They also lack other human cell lineages that could be potentially involved in cancer progression and metastasis (e.g., resident hepatocytes, osteocytes, adipocytes, nerve cells, etc.), and lack absolute efficiency of a memory-based HIS in mice. In the future, advances in the regenerative medicine field would allow for utilizing artificial human organoids and/or cells with multilineage engraftment potentials derived from tissue-restricted/induced progenitors or induced pluripotent stem (iPS) cells for generating ideal multicellular “humanized” models.

One of the important objectives of biomedical sciences is to decipher the understanding of the fundamental biological principles in order to improve clinical-pathological outcomes. It is clear that no single model is likely to recapitulate all aspects of the complex genetics and biology of human cancers; therefore, understanding the strength and limitation of each model is necessary to maximally leverage these complimentary engineered model systems to facilitate the development of improved therapeutic approaches. 3D cultures enable the identification of cellular interactions and molecular signals capable of promoting tissue regeneration and disease progression. Primary human cells or tissues can be used to guide the choice of personalized interventions, to test therapeutic schemes or to grow replacement tissues from the cells of a given patient.

The success of this approach will involve the expertise of an interdisciplinary team of scientists, clinicians, and bioengineers and require an extraordinary level of standardization; nevertheless, given the vast applications, we believe that 3D cultures represent the new frontiers of precision medicine.

## Author contributions

MB designed research, performed research, analyzed data, and wrote the manuscript. AF designed research and analyzed data. AP designed research, performed research and analyzed data. MY designed research and analyzed data. HS designed research, performed research, analyzed data, wrote the manuscript, and supervised the study.

### Conflict of interest statement

The authors declare that the research was conducted in the absence of any commercial or financial relationships that could be construed as a potential conflict of interest.

## References

[B1] Baca S. C. Prandi D. Lawrence M. S. Mosquera J. M. Romanel A. Drier Y.. (2013). Punctuated evolution of prostate cancer genomes. Cell 153, 666–677. 10.1016/j.cell.2013.03.021 23622249 PMC3690918

[B2] Bansal N. Davis S. Tereshchenko I. Budak-Alpdogan T. Zhong H. Stein M. N.. (2014). Enrichment of human prostate cancer cells with tumor initiating properties in mouse and zebrafish xenografts by differential adhesion. Prostate 74, 187–200. 10.1002/pros.22740 24154958 PMC3939797

[B3] Barbieri C. E. Bangma C. H. Bjartell A. Catto J. W. Culig Z. Grönberg H.. (2013). The mutational landscape of prostate cancer. Eur. Urol. 64, 567–576. 10.1016/j.eururo.2013.05.029 23759327 PMC4342117

[B4] Bartucci M. Patrizii M. Huselid E. Yussuf S. Bansal N. Flaherty K. (2015). Generation of single cell-derived normal, benign and cancer mini-prostates from primary patient-derived tissues, in Proceedings of the 106th Annual Meeting of the American Association for Cancer Research (Philadelphia, PA).

[B5] Berger M. F. Lawrence M. S. Demichelis F. Drier Y. Cibulskis K. Sivachenko A. Y.. (2011). The genomic complexity of primary human prostate cancer. Nature 470, 214–220. 10.1038/nature09744 21307934 PMC3075885

[B6] Billerbeck E. Labitt R. N. Vega K. Frias-Staheli N. Dorner M. Xiao J. W.. (2014). Insufficient interleukin-12 signalling favours differentiation of human CD4(+) and CD8(+) T cells into GATA-3(+) and GATA-3(+) T-bet(+) subsets in humanized mice. Immunology 143, 202–218. 10.1111/imm.12304 24766459 PMC4172137

[B7] Birgersdotter A. Sandberg R. Ernberg I. (2005). Gene expression perturbation *in vitro*–a growing case for three-dimensional (3D) culture systems. Semin. Cancer Biol. 15, 405–412. 10.1016/j.semcancer.2005.06.009 16055341

[B8] Bissell M. J. Labarge M. A. (2005). Context, tissue plasticity, and cancer: are tumor stem cells also regulated by the microenvironment? Cancer Cell 7, 17–23. 10.1016/j.ccr.2004.12.013 15652746 PMC2933216

[B9] Cassidy J. W. Caldas C. Bruna A. (2015). Maintaining tumor heterogeneity in patient-derived tumor xenografts. Cancer Res. 75, 2963–2968. 10.1158/0008-5472.CAN-15-0727 26180079 PMC4539570

[B10] Celis J. E. Gromov P. (2003). Proteomics in translational cancer research: toward an integrated approach. Cancer Cell 3, 9–15. 10.1016/S1535-6108(02)00242-8 12559171

[B11] Centenera M. M. Raj G. V. Knudsen K. E. Tilley W. D. Butler L. M. (2013). *Ex vivo* culture of human prostate tissue and drug development. Nat. Rev. Urol. 10, 483–487. 10.1038/nrurol.2013.126 23752995

[B12] Chen D. S. Mellman I. (2013). Oncology meets immunology: the cancer-immunity cycle. Immunity 39, 1–10. 10.1016/j.immuni.2013.07.012 23890059

[B14] Chong M. S. Lim J. Goh J. Sia M. W. Chan J. K. Teoh S. H. (2014). Cocultures of mesenchymal stem cells and endothelial cells as organotypic models of prostate cancer metastasis. Mol. Pharm. 11, 2126–2133. 10.1021/mp500141b 24779855

[B15] Collins A. T. Maitland N. J. (2006). Prostate cancer stem cells. Eur. J. Cancer 42, 1213–1218. 10.1016/j.ejca.2006.01.037 16632344

[B16] Cukierman E. Pankov R. Yamada K. M. (2002). Cell interactions with three-dimensional matrices. Curr. Opin. Cell Biol. 14, 633–639. 10.1016/S0955-0674(02)00364-2 12231360

[B17] Drake A. C. Chen Q. Chen J. (2012). Engineering humanized mice for improved hematopoietic reconstitution. Cell. Mol. Immunol. 9, 215–224. 10.1038/cmi.2012.6 22425741 PMC4081442

[B18] Fontugne J. Davis K. Palanisamy N. Udager A. Mehra R. McDaniel A. S.. (2016). Clonal evaluation of prostate cancer foci in biopsies with discontinuous tumor involvement by dual ERG/SPINK1 immunohistochemistry. Mod. Pathol. 29, 157–165. 10.1038/modpathol.2015.148 26743468 PMC4732921

[B19] Gao D. Vela I. Sboner A. Iaquinta P. J. Karthaus W. R. Gopalan A.. (2014). Organoid cultures derived from patients with advanced prostate cancer. Cell 159, 176–187. 10.1016/j.cell.2014.08.016 25201530 PMC4237931

[B20] Garrido-Laguna I. Uson M. Rajeshkumar N. V. Tan A. C. de Oliveira E. Karikari C.. (2012). Tumor engraftment in nude mice and enrichment in stroma- related gene pathways predict poor survival and resistance to gemcitabine in patients with pancreatic cancer. Clin. Cancer Res. 17, 5793–5800. 10.1158/1078-0432.CCR-11-0341 21742805 PMC3210576

[B21] Griffith L. G. Swartz M. A. (2006). Capturing complex 3D tissue physiology *in vitro*. Nat. Rev. Mol. Cell Biol. 7, 211–224. 10.1038/nrm1858 16496023

[B22] Gundem G. Van Loo P. Kremeyer B. Alexandrov L. B. Tubio J. M. Papaemmanuil E.. (2015). The evolutionary history of lethal metastatic prostate cancer. Nature 520, 353–357. 10.1038/nature14347 25830880 PMC4413032

[B23] Haffner M. C. Mosbruger T. Esopi D. M. Fedor H. Heaphy C. M. Walker D. A.. (2013). Tracking the clonal origin of lethal prostate cancer. J. Clin. Invest. 123, 4918–4922. 10.1172/JCI70354 24135135 PMC3809798

[B24] Hunter T. (2000). Signaling–2000 and beyond. Cell 100, 113–127. 10.1016/S0092-8674(00)81688-8 10647936

[B25] Hynds R. E. Giangreco A. (2013). Concise review: the relevance of human stem cell-derived organoid models for epithelial translational medicine. Stem Cells 31, 417–422. 10.1002/stem.1290 23203919 PMC4171682

[B26] Izumchenko E. Meir J. Bedi A. Wysocki P. T. Hoque M. Sidransky D. (2016). Patient derived xenografts as tools in pharmaceutical development. Clin. Pharmacol. Ther. 99, 612–621. 10.1002/cpt.354 26874468

[B27] Julien S. Merino-Trigo A. Lacroix L. Pocard M. Goéré D. Mariani P.. (2012). Characterization of a large panel of patient-derived tumor xenografts representing the clinical heterogeneity of human colorectal cancer. Clin. Cancer Res. 18, 5314–5328. 10.1158/1078-0432.CCR-12-0372 22825584

[B28] Kantoff P. W. Higano C. S. Shore N. D. Berger E. R. Small E. J. Penson D. F.. (2010). Sipuleucel-T immunotherapy for castration-resistant prostate cancer. N. Engl. J. Med. 363, 411–422. 10.1056/NEJMoa1001294 20818862

[B29] Lancaster M. A. Knoblich J. A. (2014). Organogenesis in a dish: modeling development and disease using organoid technologies. Science 345:1247125. 10.1126/science.1247125 25035496

[B30] Meads M. B. Gatenby R. A. Dalton W. S. (2009). Environment-mediated drug resistance: a major contributor to minimal residual disease. Nat. Rev. Cancer 9, 665–674. 10.1038/nrc2714 19693095

[B31] Mitaka T. Sato F. Mizuguchi T. Yokono T. Mochizuki Y. (1999). Reconstruction of hepatic organoid by rat small hepatocytes and hepatic nonparenchymal cells. Hepatology 29, 111–125. 10.1002/hep.510290103 9862857

[B32] Mittal D. Gubin M. M. Schreiber R. D. Smyth M. J. (2014). New insights into cancer immunoediting and its three component phases–elimination, equilibrium and escape. Curr. Opin. Immunol. 27, 16–25. 10.1016/j.coi.2014.01.004 24531241 PMC4388310

[B33] Moore K. A. Lemischka I. R. (2006). Stem cells and their niches. Science 311, 1880–1885. 10.1126/science.1110542 16574858

[B34] Nelson C. M. Bissell M. J. (2006). Of extracellular matrix, scaffolds, and signaling: tissue architecture regulates development, homeostasis, and cancer. Annu. Rev. Cell Dev. Biol. 22, 287–309. 10.1146/annurev.cellbio.22.010305.104315 16824016 PMC2933192

[B35] Postovit L. M. Costa F. F. Bischof J. M. Seftor E. A. Wen B. Seftor R. E.. (2007). The commonality of plasticity underlying multipotent tumor cells and embryonic stem cells. J. Cell. Biochem. 101, 908–917. 10.1002/jcb.21227 17177292

[B36] Pretlow T. G. Yang B. Pretlow T. P. (1995). Organ culture of benign, aging, and hyperplastic human prostate. Microsc. Res. Tech. 30, 271–281. 10.1002/jemt.1070300403 7541675

[B37] Prins G. S. Putz O. (2008). Molecular signaling pathways that regulate prostate gland development. Differentiation 76, 641–659. 10.1111/j.1432-0436.2008.00277.x 18462433 PMC2824174

[B38] Ryan D. Ren K. Wu H. (2011). Single-cell assays. Biomicrofluidics 5, 21501. 10.1063/1.3574448 21559238 PMC3089644

[B39] Sabaawy H. Bartucci M. Stein M. N. Kim I. Y. Bertino J. DiPaola R. S. (2015). Single cell patient-derived organoids for prostate cancer precision therapy, in Proceedings of the AACR-NCI-EORTC International Conference: Molecular Targets and Cancer Therapeutics (Boston, MA; Philadelphia, PA).

[B40] Sabaawy H. E. (2014). Genetic heterogeneity and clonal evolution of tumor cells and their impact on precision cancer medicine. J. Leuk. 1:1000124. 10.4172/2329-6917.1000124 24558642 PMC3927925

[B41] Sato T. Stange D. E. Ferrante M. Vries R. G. Van Es J. H. Van den Brink S.. (2011). Long-term expansion of epithelial organoids from human colon, adenoma, adenocarcinoma, and Barrett's epithelium. Gastroenterology 141, 1762–1772. 10.1053/j.gastro.2011.07.050 21889923

[B42] Sato T. Vries R. G. Snippert H. J. van de Wetering M. Barker N. Stange D. E.. (2009). Single Lgr5 stem cells build crypt-villus structures *in vitro* without a mesenchymal niche. Nature 459, 262–265. 10.1038/nature07935 19329995

[B43] Schreiber R. D. Old L. J. Smyth M. J. (2011). Cancer immunoediting: integrating immunity's roles in cancer suppression and promotion. Science 331, 1565–1570. 10.1126/science.1203486 21436444

[B44] Shamir E. R. Ewald A. J. (2014). Three-dimensional organotypic culture: experimental models of mammalian biology and disease. Nat. Rev. Mol. Cell Biol. 15, 647–664. 10.1038/nrm3873 25237826 PMC4352326

[B44a] Shultz L. D. Brehm M. A. Garcia-Martinez J. V. Greiner D. L. (2012). Humanized mice for immune system investigation: progress, promise and challenges. Nat. Rev. Immunol. 12, 786–798. 10.1038/nri.3311 23059428 PMC3749872

[B45] Siegel R. L. Miller K. D. Jemal A. (2015). Cancer statistics, 2015. CA Cancer J. Clin. 65, 5–29. 10.3322/caac.21254 25559415

[B46] Stingl J. Eaves C. J. Zandieh I. Emerman J. (2001). T. Characterization of bipotent mammary epithelial progenitor cells in normal adult human breast tissue. Breast Cancer Res. Treat. 67, 93–109. 10.1023/A:1010615124301 11519870

[B47] Wilding J. L. Bodmer W. F. (2014). Cancer cell lines for drug discovery and development. Cancer Res. 74, 2377–2384. 10.1158/0008-5472.CAN-13-2971 24717177

[B48] Wilson H. V. (1907). A new method by which sponges may be artificially reared. Science 25, 912–915. 10.1126/science.25.649.912 17842577

[B49] Yamada K. M. Cukierman E. (2007). Modeling tissue morphogenesis and cancer in 3D. Cell 130, 601–610. 10.1016/j.cell.2007.08.006 17719539

[B50] Yin X. Mead B. E. Safaee H. Langer R. Karp J. M. Levy O. (2016). Engineering stem cell organoids. Cell Stem Cell 18, 25–38. 10.1016/j.stem.2015.12.005 26748754 PMC4728053

