# Corrigendum: Personalized Medicine Approaches in Prostate Cancer Employing Patient Derived 3D Organoids and Humanized Mice

**DOI:** 10.3389/fcell.2016.00074

**Published:** 2016-07-11

**Authors:** Monica Bartucci, Anna C. Ferrari, Isaac Yi Kim, Alexander Ploss, Martin Yarmush, Hatem E. Sabaawy

**Affiliations:** ^1^Rutgers Cancer Institute of New Jersey, Rutgers UniversityNew Brunswick, NJ, USA; ^2^Department of Molecular Biology, Princeton UniversityPrinceton, NJ, USA; ^3^Center for Engineering in Medicine, Shriners Hospitals for Children and Department of Surgery, Massachusetts General Hospital, Harvard Medical SchoolBoston, MA, USA; ^4^Department of Biomedical Engineering, Rutgers UniversityNew Brunswick, NJ, USA; ^5^Department of Medicine, Rutgers Biomedical and Health Sciences (RBHS)-Robert Wood Johnson Medical School, Rutgers UniversityNew Brunswick, NJ, USA

**Keywords:** organoids, prostate cancer, precision medicine, precision therapeutics

Reason for Corrigendum:

In the original Perspective article there was an error in the mouse strain described in Figure 2G its legend, and the corresponding reference.

In the first paragraph of the section Reconstitution of Mice with a Humanized Immune System, the reference (Chen et al., 2009) has been replaced with (Shultz et al., 2012).

The correct versions of Figure 2, its legend, and the first paragraph of the section Reconstitution of Mice with a Humanized Immune System appear below. The authors sincerely apologize for the error. This error does not change the scientific conclusions of the Perspective in any way.

**Figure 2:**
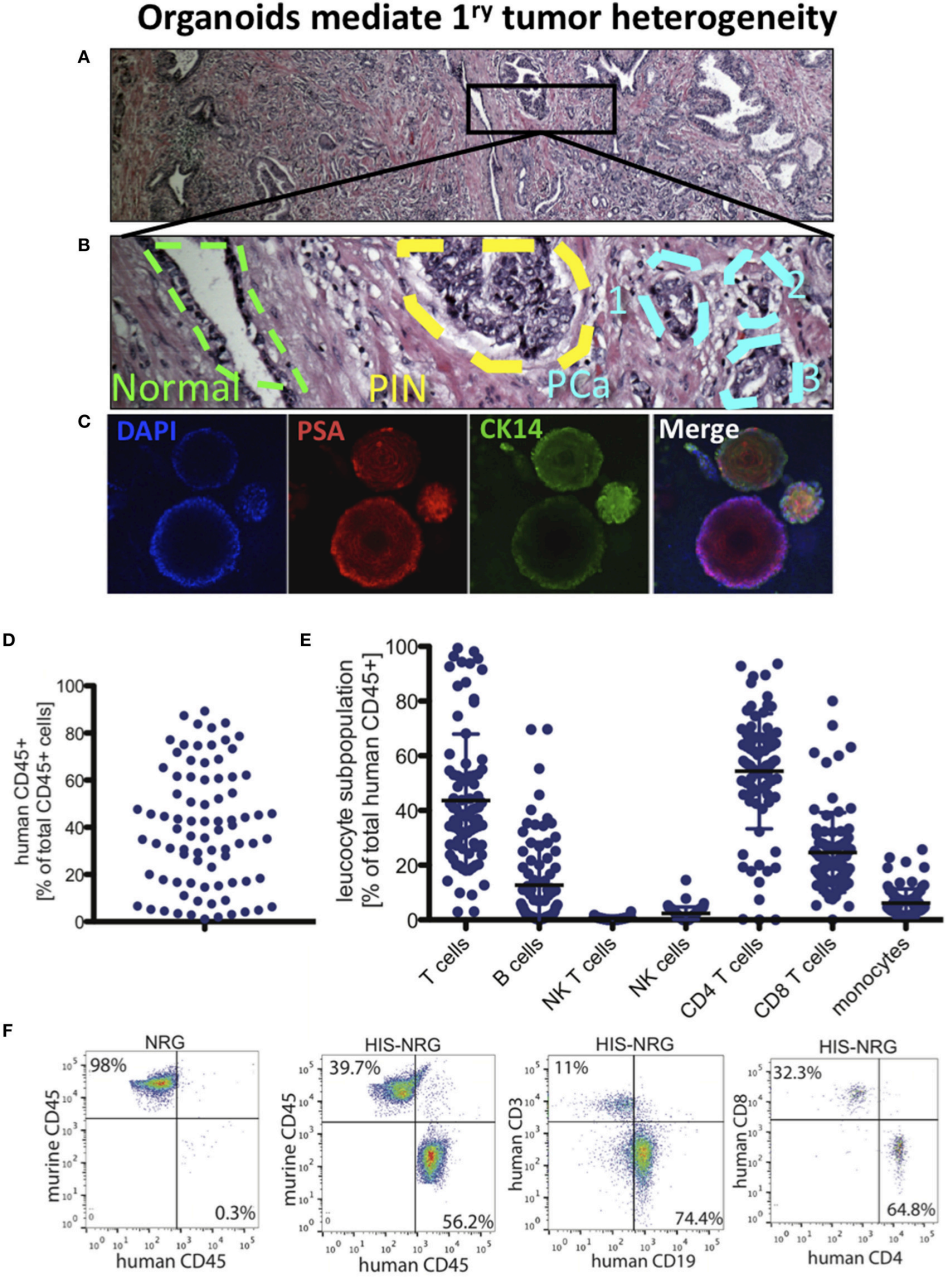
**PCa organoids to model tumor heterogeneity and develop immunotherapy in humanized mice. (A)** H&E of RP section from a PCa patient shown in 4x. **(B)** The outlined area in **(A)** is displayed in 200x, showing the outline of benign prostate gland (green), prostatic intraepithelial neoplasia (PIN) region (yellow), and a three foci region of PCa (Blue). **(C)** Single-cell organoids reflect the heterogeneity in primary PCa. Immunofluorescence (IF) images show DAPI as nuclear staining, PSA (center region), CK14 (in cells lacking PSA staining, i.e., transit amplifying cells). Multiple organoids derived from the same patient's PCa expressing PSA and CK14 (right), low and localized (top) and low/negative (bottom). **(D)** Human immune system (HIS) reconstitution in NRG HIS. Fraction of human CD45+ cells of total CD45+ cells detected in the HSC transplanted NRG mice. **(E)** Indicated leukocyte subpopulations were determined by FACS analysis of PBMC in NRG mice. **(F)** Indicated immune cell subpopulations in NRG and HIS-NRG mice are shown as dot plots.

The original article has been updated.

## Reconstitution of mice with a humanized immune system

Humanized mice are immunodeficient animals engrafted with human hematopoietic stem cells (HSCs) that give rise to various lineages of human blood cells throughout the life of the mouse (Drake et al., 2012). By simultaneously humanizing the immune system of recipient animals and challenging them with implanted human tumor cells in prostate organoids, the interactions between human immune cells and tumor cells can be interrogated (Chen and Mellman, 2013). Mice engrafted with components of a human immune system (HIS) are routinely generated by engrafting human HSCs isolated from human fetal liver (HFL), BM, or cord blood into highly immunodeficient mouse strains, such as NOD Rag1^−∕−^ IL2Rα^null^ (NRG) mice, NOD SCID IL2Rα^null^ (NSG) or Balb/C Rag2^−∕−^ IL2Rα^null^ (BRG), that support better human hematopoietic cell engraftment (Figures 2D–F). We have pursued improved strategies to enhance the human immune cell reconstitution and function in humanized mice (Billerbeck et al., 2014). A variety of other strategies are also being pursued, including, but are not limited to: the expression of human orthologs of non-redundant cytokines with limited biological cross-reactivity to foster the development of human immune cell lineages which currently do not develop efficiently in conventional humanized mice; expression of human MHC in the absence of mouse MHC to ensure faithful presentation of self- and virally-derived peptides to human T-cells and to reduce graft-versus-host-disease; co-transplantation of HSC donor-matched human thymic cortical epithelium to facilitate proper T-cell selection; the improvement of lymphoid architectural organization, especially in the spleen and lymph-nodes, to allow for adequate T- and B-cell priming; genetic replacement of non-compatible immune cell receptors and chemokines expressed on non-hematopoietically derived cells to improve immune functions such as immune cell trafficking; and the introduction of a human microbiome to account for the effects of species-specific commensals on the immune system (reviewed in Shultz et al., 2012). We are now poised to reconstitute mice with a HIS from PCa patients along with their prostate organoids to examine the personalized immune responses against PCa cells in the presence or absence of PCa therapy to overcome resistance and provide new approaches for cancer therapy.

## Author contributions

MB designed research, performed research, analyzed data, and wrote the manuscript. AF designed research and analyzed data. IK designed research and analyzed data. AP designed research, performed research and analyzed data. MY designed research and analyzed data. HS designed research, performed research, analyzed data, wrote the manuscript, and supervised the study.

## Funding

This project was supported by Rutgers Cancer Institute of New Jersey (Pilot Grant to HS), National Cancer Institute (P30 CA072720 to AF, IK, AP, MY, and HS), and New Jersey Health Foundation Innovation Award (ISFP 7-16 to HS).

### Conflict of interest statement

The authors declare that the research was conducted in the absence of any commercial or financial relationships that could be construed as a potential conflict of interest.
